# South American Validation of a Survey to Assess Eco-Anxiety in Adults (ECO-ANS-LATAM)

**DOI:** 10.3390/jcm13082398

**Published:** 2024-04-20

**Authors:** Christian R. Mejia, Aldo Alvarez-Risco, David A. Vizcardo, Luzmila Sedano-Ochoa, Maria Fe Medina Rivera, Claudia Shimabukuro Jaramillo, Jamil Cedillo-Balcázar, Oscar Mamani-Benito, Renzo Felipe Carranza Esteban, Jose Armada, Milward Ubillus, Shyla Del-Aguila-Arcentales, Neal M. Davies, Jaime A. Yáñez

**Affiliations:** 1 Asociación Médica de Investigación y Servicios en Salud, Lima 15304, Peru; christian.mejia.md@gmail.com; 2 Facultad de Medicina, Universidad Peruana de Ciencias Aplicadas, Lima 15023, Peru; david.arturoalba@hotmail.com (D.A.V.); luzmilasedanoochoa@gmail.com (L.S.-O.); mafemedd@gmail.com (M.F.M.R.); claudiashima54@gmail.com (C.S.J.); 3 Facultad de Administración y Negocios, Universidad Tecnológica del Perú, Lima 150101, Peru; 4 Carrera de Medicina, Facultad de Ciencias en la Salud, Universidad Técnica Particular de Loja, Loja 110107, Ecuador; jamcbmail@gmail.com; 5 Facultad de Ciencias de la Salud, Universidad Señor de Sipán, Chiclayo 14000, Peru; mamanibe@uss.edu.pe; 6 Carrera de Psicología, Facultad de Ciencias de la Salud, Universidad San Ignacio de Loyola, Lima 15023, Peru; rcarranza@usil.edu.pe; 7 Faculty of Business Sciences, Universidad Continental, Huancayo 12101, Peru; jarmada@continental.edu.pe; 8 Universidad de Huánuco, Huánuco 10003, Peru; milward.ubillus@udh.edu.pe; 9 Escuela de Posgrado, Universidad San Ignacio de Loyola, Lima 15023, Peru; sdelaguila@usil.edu.pe; 10 Faculty of Pharmacy and Pharmaceutical Sciences, University of Alberta, Edmonton, AB T6G 1H9, Canada; ndavies@ualberta.ca; 11 Facultad de Educación, Carrera de Educación y Gestión del Aprendizaje, Universidad Peruana de Ciencias Aplicadas, Lima 15023, Peru; pcedjyan@upc.edu.pe

**Keywords:** anxiety, climate change, validation, instrumental study, South America

## Abstract

**Background**: climate change is a reality, and more and more people are becoming aware of this global problem, which has generated anxiety in some populations. To validate a short survey to assess eco-anxiety in adults in South America. **Methods**: It is an instrumental study, and the validation was based on a previous survey, which had six questions and was generated by 217 respondents in the USA in 2021. These questions were subjected to a validation process with expert judgment, pilot and application, and then statistics were obtained. It was validated with 1907 people in six countries in South America, where the mean, standard deviation, skewness and kurtosis were adequate. **Results**: The initial confirmatory factorial model obtained unsatisfactory goodness-of-fit indices, so the indices were modified through a re-specification, where two items were eliminated, after which adequate values were obtained (χ^2^ = 22.34, df = 2, *p* = 0.00; RMR = 0.020; GFI = 0.990; CFI = 0.990; TLI = 0.990; and RMSEA = 0.070). Finally, the overall Cronbach’s α was calculated to be 0.88 (95% CI = 0.86–0.89). **Conclusions**: The test was validated in a large South American population and found that only four questions can efficiently measure anxiety about the effects of climate change. The instrument can be used with other tests to screen different age groups, ethnicities and realities.

## 1. Introduction

For some decades now, scientific results have been emerging about imminent climate change that the entire planet is suffering, mainly because the greenhouse effect traps energy, and this gradually increases the global temperature [1], which as a whole generates a warming of the oceans and melting of glaciers, among many other effects [2], which were not considered much at first; however, climate change gradually began to gain the interest of scientists and even the general population, which precipitated the development of summits, treaties, pacts and other actions to curb climate change. Some of the most important meetings have been held since 1972 [3], and since then, there have been many such meetings, with the last one taking place in Egypt in 2022 [4]. It is in this context that more and more people and groups are becoming aware of and concerned about the deterioration of the climate and the repercussions it may have on a global scale; some research has even shown that the population is becoming increasingly aware of climate change and has a significant perception of the risks that may be associated with this phenomenon [5]. It is important to consider that understanding the concept of climate change depends on the culture of the country where the measurement is carried out. Although there is consensus on several components, it is necessary to make this comment to consider the validation processes between countries.

Assessing anxiety within a community is crucial for several reasons. Firstly, anxiety is a common disorder that can have a negative impact on an individual’s quality of life and their ability to function in society. By identifying anxiety within a community, appropriate resources and support services can be provided to help those who suffer from it. Anxiety affects not only individuals but also their families and communities in general, as it can have an impact on interpersonal relationships, work performance and social participation. Also, anxiety is not always easy to recognize and can manifest itself in various ways, making its assessment crucial to detect it and provide early interventions. Then, understanding the prevalence and factors associated with anxiety in a community can inform public health policies and prevention programs. Overall, assessing anxiety within a community is vital to promote individual and collective well-being, as well as to develop effective strategies to address this disorder in an appropriate and timely manner. There are some questionnaires in the literature, but they do not specifically measure anxiety due to ecological issues, such as the impact generated on concentration, sleep, abilities and work, which are components that anxiety affects. The present study will specifically address eco-anxiety.

For this reason, instruments have been developed to measure anxiety about climate change and other related attitudes, an important one being the survey carried out in the United States in 2021, in which 217 adults were surveyed to measure their anxiety about climate change and other aspects related to it [6]. This tool was chosen because it was noted that the previous validation process in the reference article was carried out correctly and, therefore, reliably, allowing the questionnaire items to be considered. However, although the other issues that were evaluated are essential, short instruments should be developed that directly and forcefully measure anxiety generated by climate change because multiple systematic reviews and meta-analyses show that the population already has a degree of anxiety, depression and other disorders in the mental sphere [7,8]. Furthermore, this must be performed in important populations, such as South America, where the COVID-19 pandemic showed us to have governments that are not prepared to face global problems due to the political crises that each of the countries in the region are going through, and which generate an environment conducive to the progressive and substantial deterioration of the mental health of its population [9,10]. For all these reasons, the research aimed to validate a short survey to assess eco-anxiety in adults in South America.

## 2. Materials and Methods

Through an instrumental study, 1907 people were surveyed in six South American countries: 713 people in Peru, 465 in Colombia, 304 in Ecuador, 222 in Bolivia, 143 in Paraguay and 60 in Argentina. The survey population consisted of 1229 women (64.5%), with a median age of 23 (interquartile range: 21–29 years). Of these women, 69.6% had a university degree and 80.1% were unmarried. To know how many people to survey, the consensus for this type of design was followed, establishing that a minimum of 20 people should be surveyed for each evaluation question. However, since it was a multicenter investigation, we sought to obtain the greatest amount of data, so with 1907 participants, it was possible to have an average of 317.83 participants per question. At the beginning of the research, the project was carried out and subsequently submitted for review to the ethics committee of the Peruvian University of Applied Sciences in Peru, which gave its authorization (approval code: FCS-SCEI-109-03-22). A thorough review of the scientific literature played a pivotal role by identifying pertinent previous studies and questionnaires employed in analogous contexts. The proposed scale was based on the previously mentioned survey (6), which was developed and validated in the population of the United States of America. Special emphasis was placed on the cultural and linguistic adaptation of the selected items to ensure their applicability in the Latin American region, so we took only six questions that inquired about anxiety caused by climate change that our planet is undergoing; the other sixteen questions were not taken into account, as they addressed other aspects that were not of interest for this research. It is important to mention that the six questions had five possible answers (strongly disagree, disagree, indifferent, agree and strongly agree). Therefore, these questions were the basis for the experts’ evaluation.

In the expert validation step, eight health professionals from the countries where the surveys were conducted were asked to evaluate the relevance, representativeness and clarity of each of the six questions; they could even provide comments (some provided them, but it was more in the aspect of the global context, so they were not officially incorporated); in each case they were asked to give a mark for each question and each type of assessment, and in each case the marks could range from zero (where they said it was not at all clear, relevant or representative) to three points (where it was apparent, relevant or representative). The results of this stage are shown in the preliminary data analysis section.

Then we proceeded to carry out a pilot with 30 people who would be similar to those surveyed in the next stage; this served to determine the approximate time of resolution so that they could send us doubts about the wording and understanding of the questions, among other things; this step was completed as expected and with that we proceeded to the last step. For the last part, the questions were uploaded to a Google Forms form, where the link was sent to the respondents in the six countries already mentioned. No sensitive data was asked, the survey was completely anonymous, and their rights and the research purpose were explained to them. The survey used in this study was adapted for virtual distribution to broaden its reach across several countries. It was circulated via social networks (WhatsApp and Facebook) from the third week of April 2022 to the first week of September 2022. The main objective was to engage individuals aged 18 and above in Peru, Colombia, Ecuador, Bolivia, Paraguay and Argentina. To facilitate this outreach, the researchers collaborated with healthcare professionals, ensuring confidentiality and seeking their support in sharing the survey. Before completing the survey, respondents were asked if they wished to participate; those who agreed were asked to choose the option “I agree” and less than 30 people chose the option “I do not agree to participate in the survey”. Those who agreed to be surveyed went on to complete the survey. Once the survey period was over, the responses were downloaded into the Microsoft Excel program (version 2019), where quality control of the data was carried out and data were filtered according to the selection criteria in order to proceed to the psychometric analysis.

### Psychometric Analysis of the Data

A descriptive analysis was carried out to begin the analysis, obtaining the mean scores, standard deviations, skewness and kurtosis for each of the six questions of the initial scale (Table 1); this was performed using the FACTOR Analysis program (version 11.05). The kurtosis and skewness scores corroborated that the sample obtained had a normal distribution by obtaining scores below ±1.5 [11]. For the AFE, the Kaiser–Meyer–Olkin (KMO) coefficient and Bartlett’s test were obtained, which is where the unweighted least squares estimation method was considered, using Promin rotation [12]. We then proceeded with the Confirmatory Factor Analysis (CFA), where we used the AMOS statistical program (version 21), with which we performed structural equation modeling (SEM), obtained the goodness-of-fit index (GFI), the Tucker–Lewis Index (TLI), the comparative fit index (CFI), the root mean square error of approximation (RMSEA) and the root mean square error index (RMR). For the evaluation of each of these indices/values, the criteria proposed by Hu and Bentler [13] were followed, and they stated that the GFI, TLI and CFI should be greater than 0.9 and the RMSEA less than 0.08. Finally, the reliability of the scale was calculated through Cronbach’s Alpha coefficient (α) using the SPSS statistical program (version 25.0), as well as the McDonald’s Omega coefficient (ω) using the Jamovi program version 2.3.18.

## 3. Results

### 3.1. Preliminary Analysis of the Items

In the analysis of the experts’ answers, we found Aiken’s V values for relevance (question 1: 0.67; question 2: 0.67; question 3: 0.75; question 4: 0.75; question 5: 0.58; and question 6: 0.71), representativeness (question 1: 0.63; question 2: 0.71; question 3: 0.79; question 4: 0.83; question 5: 0.67; and question 6: 0.71) and clarity (question 1: 0.63; question 2: 0.67; question 3: 0.75; question 4: 0.75; question 5: 0.63; and question 6: 0.86). Some of these values were below what was expected; however, the recommendation was that the six questions move on to the following stages because they were already from a previously validated instrument, so how they performed in the pilot phase and the large population surveyed were to be assessed. Table 1 shows the mean, standard deviation, skewness and kurtosis values for the six initial scale items validated in the United States of America. Item 4 has the highest mean score (mean = 3.26) and the highest dispersion (standard deviation = 1.29). The skewness and kurtosis of the six items of the scale do not exceed the range > ±1.5 [14].

### 3.2. Confirmatory Factor Analysis (CFA)

Previous evidence was considered to verify the internal structure, so a CFA with a unidimensional structure was carried out; the results of the original model reported unsatisfactory goodness-of-fit indices. For that, through the index modification technique, a re-specification was carried out, where items 4 and 6 were eliminated, and a satisfactory factorial structure model was obtained (Table 2).

**Figure 1:**
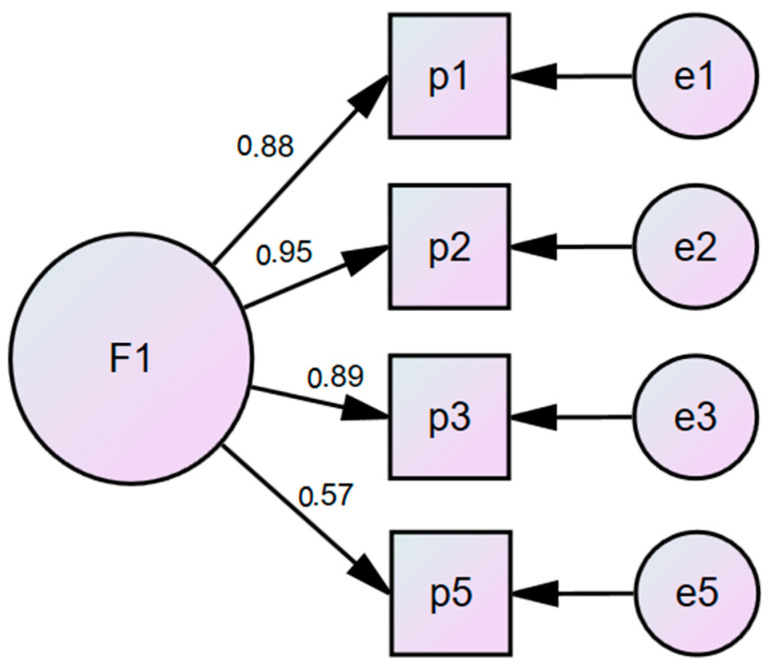
ECO-ANS-LATAM scale model 1.

Figure 1 shows that the four-item model can be grouped into a single factor, having adequate values (χ^2^ = 22.34, df = 2, *p* = 0.00; RMR = 0.020; GFI = 0.990; CFI = 0.990; TLI = 0.990; and RMSEA = 0.070).

### 3.3. Reliability and Final Test

Finally, the reliability of the ECO-ANS-LATAM scale was calculated, obtaining an acceptable Cronbach’s Alpha coefficient (overall α = 0.88; 95% CI = 0.86–0.89) and a McDonald’s Omega coefficient (ω = 0.87). Therefore, if one wants to use the final ECO-ANS-LATAM scale, one has to consider the four questions in Table 3, each of which can have five possible answers (strongly disagree, disagree, indifferent, agree and strongly agree). To determine who has the most eco-anxiety, it is suggested that tercile analysis be used, whereby for each response, respondents give one point each time they strongly disagree and up to five points each time they strongly agree. Thus, the range of scores could be between four to twenty possible points; it is only a matter of ordering the scores, and all those in the top tercile of the scores obtained can be considered as having the most anxiety about climate change and those with scores in the middle tercile or the bottom tercile are those who do not have the most anxiety about climate change (thus obtaining a dichotomous variable).

## 4. Discussion

Climate change is an issue that is here to stay, and much depends on what governments decide and how they act in the coming years. Every year, at the Conference of the Parties (COP), countries from various parts of the world convene to establish guidelines aimed at reducing and adapting to the effects of climate change [15]. For this reason, this topic must now be properly addressed, as a topical issue with repercussions in the mental sphere. Several studies claim that during heat waves, psychiatric visits and hospitalizations increase [16]. In other countries, this has been seen some years ago, and the mental sphere, the repercussions it generates and even other things it is associated with have started to be evaluated. Regarding a study conducted by Amruta Nori, an increase in temperature of one degree Celsius was associated with heightened anxiety and mood disorders, potentially impacting physical health as well (Newtral 2023). Across different nations, this correlation has been observed over several years, prompting comprehensive assessments of its impact on mental health and exploration of the various ramifications associated with this phenomenon.

For example, a study in 32 countries found that much of the population had negative emotional responses to climate change [17]. Similarly, in Norway, the most frequently mentioned emotions were fear, sadness, anger and hopelessness [18], which is why it was decided to validate the scale that measures eco-anxiety in the Latin American context, knowing that in our region there are already previous studies that report significant levels of anxiety, such as the study where the prevalence of mental health symptoms was analyzed based on 341 studies, with a total of almost two million participants from Latin America and elsewhere, where 32% had mental health symptoms that were worse than the general or common ones; among the most prevalent and prominent psychological symptoms in the sample, it was observed that a significant percentage experienced anxiety, at 29%, closely followed by depression, which registered at 27% of the sample. Additionally, a significant 25% of the participants reported experiencing high levels of anxiety, emphasizing the importance and relevance of these psychological conditions in the studied population.

In addition, people in less developed countries suffered less than those in emerging or developing countries [19]. Another article reporting high levels of anxiety in Latin America evaluated a total of 62 studies, with almost 200,000 participants, and found a prevalence of anxiety of 35%, with a higher prevalence of mental health symptoms in South America (36%), at least compared to Central America (28%) [20]. However, in the literature, there are no massive studies with large samples on eco-anxiety, which could help us better understand this disorder, which is already being reported in other parts of the world. The validation of the proposed scale arises from the need to fill a significant gap in the understanding of eco-anxiety in the Latin American context. Despite existing studies in the region, the validation is justified by the complexity of eco-anxiety, which can vary substantially in cultural and socioeconomic terms. This initiative brings innovation to the field by providing a specific and validated tool that seeks to capture unique dimensions of environmental anxiety, incorporating factors such as socio-economic particularities and local impacts of climate change in Latin America. The validation not only ensures cultural relevance but also contributes to new insights, enriching the global literature on eco-anxiety and offering a more comprehensive perspective on the psychological impacts of climate change in the region.

For this reason, this would be the first scale for adults in Latin America, based on rigorous validation and with the opinion of many experts and respondents in six of the most important Spanish-speaking countries in the region. The first three questions address the direct impact of not being able to concentrate, and the impact on work activities, academic activities or even sleep. These questions are important because they convey vital aspects of life, and previous anxiety scales also assess these aspects, such as the “Hogg Eco-Anxiety Scale” proposed in 2021, which talks about the negative impact of climate change and how this can lead to stress, anxiety and even depression [21]. In the “Climate Change Worry Scale”, very similar topics to the previously mentioned scale were evaluated [22]. But now, with this summarized scale, the compiled information is simplified, which would facilitate its analysis and comprehension, as well as effective communication with other healthcare professionals and researchers. Moreover, it will allow for tracking over time to identify changes, and the gathered information could even be utilized for other assessments that evaluate other important aspects of mental health.

Finally, a final question addresses damage to an essential place by climate change, which is increasingly observed as environmental degradation progresses and more places are affected. For example, some of the most recent reports of areas affected by climate change in South America mention that in contrast to the rest of the world, in Latin America and the Caribbean, greenhouse gas emissions are mainly produced by the agricultural sector, affecting the region mainly in the following areas: Andean glaciers due to warming, hydrological basins due to changes in rainfall patterns, tropical rainforests, ecosystem integrity and biodiversity [23,24]. Moreover, the damage caused by climate change and human activity has already produced permanent damage. An example is the Atescatempa lagoon, located in Guatemala, which suffered severe droughts since 2018, and the lake is now completely dry, affecting the town’s fishing and tourism industries. Another lake that went through the same process was Poopó, located in Bolivia, which, due to climate change, disappeared and dried up in just two years due to the melting of its glaciers and the diversion of its tributaries [25,26], which is why this fourth question becomes increasingly important as the years go by, with more and more repercussions being seen in different areas and regions.

The study has the limitation that it is impossible to extrapolate the results entirely to the countries where the survey was taken because the sampling was non-random. Additionally, there is a limitation in the sample characteristics as they mostly consist of young adults, with a significant percentage being women and respondents possessing higher education. This composition might have impacted the generalizability of the findings to a more diverse population concerning age, gender and educational level. However, most validation studies do not carry out this type of sampling, so they try to have a large sample; our study has the criterion of having a minimum of 20 respondents for each question that is validated and our results present more than 300 respondents for each original question, in six significant countries.

It is recommended that future research look at psychometric properties in their own populations, apply the scale in various samples, combine it with other factors that could be intervening, and make inferential designs.

## 5. Conclusions

For all these reasons, it is thought that the validated test can be helpful in the Latin American context, where the populations are very similar in culture, idiosyncrasy and even socio-political context. For all these reasons, it is concluded that an instrument was validated to evaluate eco-anxiety in a determined population in Latin America, where four questions were left with good results in the evaluations of the experts and the others that were carried out.

## Figures and Tables

**Table 1:** Mean, standard deviation, skewness and kurtosis of the ECO-ANS-LATAM scale.

Measures	Item 1	Item 2	Item 3	Item 4	Item 5	Item 6
Mean	2.61	2.42	2.42	3.26	3.03	3.16
Standard deviation	1.152	1.116	1.129	1.299	1.280	1.264
Skewness	0.132	0.247	0.266	−0.457	−0.208	−0.352
Kurtosis	−0.832	−0.795	−0.807	−0.919	−1.061	−0.950

**Table 2:** Goodness-of-fit indices of the ECO-ANS-LATAM scale.

The Goodness-of-Fit Index	Original	Model 1
	(6 Items)	(4 Items)
CMIN	831.72	22.34
DF	65	2
P	0.000	0.000
CMIN/DF	92.41	11.17
RMR	0.150	0.020
GFI	0.850	0.990
CFI	0.890	0.990
TLI	0.820	0.990
RMSEA	0.220	0.070

**Table 3:** Final ECO-ANS-LATAM scale post-confirmatory factor analysis and other psychometric tests.

Questions	Strongly Disagree	Disagree	Indifferent	Agree	Strongly Agree
Thinking about climate change is making it hard for me to concentrate.					
Thinking about climate change makes it hard for me to sleep.					
My concern about climate change interferes with my ability to work or with my academic activities.					
Climate change has affected a place that is important to me.					

## Data Availability

The data are available on request.
